# Toughness amplification in copper/epoxy joints through pulsed laser micro-machined interface heterogeneities

**DOI:** 10.1038/s41598-017-16471-6

**Published:** 2017-11-27

**Authors:** Edwin Hernandez, Marco Alfano, Ditho Pulungan, Gilles Lubineau

**Affiliations:** 1 King Abdullah University of Science and Technology (KAUST), Physical Science and Engineering Division, COHMAS Laboratory, Thuwal, 23955-6900 Saudi Arabia; 2 Department of Mechanical, Energy and Management Engineering (DIMEG), University of Calabria, Via P. Bucci 44C, 87036 Rende, CS Italy

## Abstract

This work addresses the mechanics of debonding along copper/epoxy joints featuring patterned interfaces. Engineered surface heterogeneities with enhanced adhesion properties are generated through pulsed laser irradiation. Peel tests are carried out to ascertain the effect of patterns shape and area fraction on the mechanical response. Experimental results are evaluated with the support of three-dimensional finite element simulations based on the use of cohesive surfaces. Results discussion is largely framed in terms of effective peel force and energy absorbed to sever the samples. It is shown that surface heterogeneities act as sites of potential crack pinning able to trigger crack initiation, propagation and arrest. Surface patterns ultimately enable a remarkable increase in the effective peel force and dissipated energy with respect to baseline homogeneous sanded interface.

## Introduction

Interfaces play a significant role on the overall mechanical performance of bonded structures in aerospace, electronics, construction and energy industries^1–6^. There has been a tremendous research effort in the area of surface modification strategies to improve interfacial adhesion in multi-material components and structures. While a significant body of published literature elucidated adhesion at homogeneous interfaces^7–10^, recent works focused on the effect of surface patterning (*e*.*g*. texturing) and demonstrated the beneficial effect on the overall joint strength and bond toughness^11–16^.

Surface modification strategies which enable the generation of surface patterns include *bottom-up* and *top-down* approaches. Bottom-up approaches include chemical or physical deposition of surface coatings and/or the generation of surface patterns where the patterning scale being possible is as small as the nanoscale level. Chan *et al*.^17^ analyzed the effect of surface patterns generated by means of template assisted silane treatments and conventional UV lithography. The results indicated an improved peel strength of soft elastomeric/glass interfaces. Guan *et al*.^18^ employed physical vapor deposition for the fabrication of a patterned copper layer on aluminum substrates. Mechanical tests have shown a strong effect on front propagation and an overall increase in the work of separation. Xia *et al*.^12–14^ analyzed the peeling behavior of polyester thin films from polydimetylsiloxane (PDMS) substrates and demonstrated that the proper arrangement of pinning sites with large adhesion energy (*i*.*e*. ink pattern on PDMS) can optimize the effective resistance of the system at the macro-scale. Cai and Dauskardt^15^ increased the adhesion of organic PMMA to silicon oxide thin films in diffusion barriers by means of interfacial nanostructures (*i*.*e*. cylindrical pillars) obtained through standard lithographic techniques. Mode I delamination tests indicated that the improvement was associated to pillars pullout. In similar fashion, Su *et al*.^16^ addressed the strength of PEO electrolyte/V_2_ O_5_ electrode interface in solid state lithium-ion batteries and analyzed the effect of pillars across the interface. Crack shielding arising from the pillars bending deformation was shown to decrease the stresses at the delamination front and resulted in increased overall interfacial peeling resistance.

*Top-down* approaches, unlike the bottom-up ones discussed above, are based on material removal from an initial surface and are potentially much flexible and amenable to large-scale applications. The dimensional capability depends on the tool used to modify the surface. Chung and Chaudhury^19^ analyzed crack propagation along silicone elastomer small-scale patterned thin films. Lateral, longitudinal and crosswise interfacial incisions were made using a sharp razor blade. They demonstrated that crack blunting induced by the incisions could lead to substantial adhesion improvements. Interfacial debonding occurred in an intermittent manner involving sequential events of initiation, propagation and arrest. Noori *et al*.^20^ employed grinding and knurling mechanical pre-treatments in order to tailor the adhesion at the interface between steel substrates and polymeric thin films. The results demonstrated that surface patterns enabled an increased plastic energy dissipation and an higher peeling resistance. Cordisco *et al*.^21^ analyzed failure along sinusoidal interfaces fabricated through wire EDM. The results indicated delayed crack initiation, intermittent crack propagation and an improved toughness because of the increase area available for bonding. More recently, Maloney and Fleck^22^ analyzed the response of architected adhesive joints. Metal substrates with square-wave patterned interfaces, obtained using water-jet cutting, were bonded with an adhesive. Their work demonstrated that, compared to the baseline planar interface, the patterns modify the development of damage within the adhesive layer and lead to increased energy dissipation.

Although much labor intensive, molding techniques have been also deployed on polymeric substrates. Pendergraph *et al*.^23^ analyzed the adhesion of elastomeric fabric composites through the use of topographical surface patterns and observed an increased shear strength with respect to the flat baseline interface. Yukimoto *et al*.^24^ generated step-shaped micro-patterns on CFRP pre-pregs using in-mold surface preparation. Mixed mode fracture tests carried out on adhesive bonded joints highlighted the efficiency of the patterns in elevating the fracture toughness. Matsuzaki *et al*.^25^ developed a roll-imprinting process to introduce mechanical interlocking through micro-patterns on polypropylene plates. An increased strength, as determined in butt joint tests, demonstrated the effectiveness of the method.

The analysis of previous related works highlights the beneficial effect of either planar or non-planar surface patterns on interfacial strength and bond toughness. The aim of this study is to extend the scope of these works by using an alternative top-down patterning technique, which leverages on the use of pulsed laser irradiation. Pulsed laser sources can induce significant melting, re-solidification and vaporization of the target material thereby enabling the simultaneous modification of surface topography and chemistry^26^. Moreover, current lasers are provided with high precision beam positioning systems which ensure the ability to engrave surface patterns with accurate geometrical control and, as a result, to generate interfaces with heterogeneous surface properties. It follows that unlike previous methods (*e*.*g*. template assisted treatments, machining and molding), laser irradiation may allow the tailored introduction of local toughening sites across the interface. However, there is a need to unravel the relationships between the shape and area fraction of laser treated material and the corresponding mechanical behaviour. Here we analyze the effect of laser micro-machined planar patterns with different shape and area fractions on the bond toughness of CuZn40/epoxy joints. This is an inherently brittle material system which entails scientific and engineering interest because of its widespread use for photovoltaics, flexible electronics, integrated circuits, to list a few^2,10,27–29^. Laser process effectiveness is strongly related to the characteristics of the laser beam, *e*.*g*. laser wavelength, pulse energy and duration. From this standpoint, nanosecond pulsed fiber lasers have been successfully used to machine several engineering materials (*e*.*g*. aluminum, steel, copper, titanium, carbon fiber reinforced laminates) and have been proven to deliver surfaces which enable efficient adhesive bonding and increased fracture toughness^7–9,30^. In the present work, surface patterns were engraved on copper through nano-second pulsed Yb-fiber laser irradiation. Substrates were subsequently bonded with structural epoxy adhesive and loaded under peel in a displacement-controlled experiment. The results discussion is largely framed in terms of effective peel force and absorbed energy needed to sever the sample. Finally, the mechanistic analysis of crack growth across the patterned interfaces required an associated computational effort. We propose herein an approach based on the use of 3D finite element simulations with cohesive surface elements.

## Results

### Local morphology and chemistry of patterned interfaces

**Figure 1:**
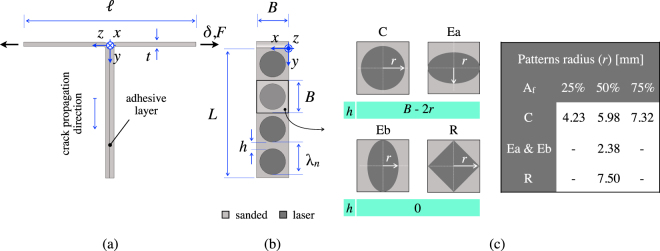
Schematic depiction of the (**a**) epoxy bonded T-joint and (**b**) corresponding patterned interface featuring the relevant geometrical key parameters (*L* = 60 mm; *B* = 15 mm; *λ*_*n*_ = 15 mm; *t* = 0.5 mm; $\ell$ = 80 mm). A thin epoxy layer (thickness 250 *μ*m) supplied the resisting forces during mechanical tests. The T-joint was pulled apart at one end at a displacement rate of 5 mm/min. (**c**) Schematics of the patterns and corresponding radius (*r*). Laser processing was carried out in the *x*-direction. The distance *h* quoted in the figure indicates the minimum distance between the outmost points of two laser treated areas.

Mechanical tests were carried out using the T-peel test coupon^31^ whose overall dimensions are given in Fig. 1. Four pattern types were engraved over the baseline sanded copper substrates represented by arrays of circles (C), ellipses (Ea and Eb) and rhombs (R). The radius *r* of each treated area as a function of the area fraction is given in Fig. 1(c). The local replacement of a weak sanded interface by one of much stronger adhesion enables the introduction of crack pinning sites. The area fraction of laser treated material (A_*f*_) was equal to 50%. However, two distinct sets of samples of type C were also included in the analysis to ascertain the effect of A_*f*_, *i*.*e*. 25% and 75%. The obtained patterned interfaces feature a periodic variation of both surface topography and chemistry which were investigated by means of XPS and SEM analyses.

**Figure 2:**
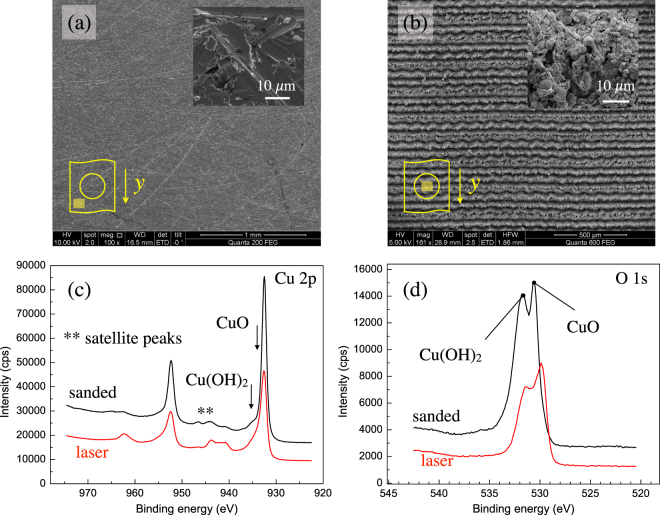
SEM images of (**a**) sanded and (**b**) laser treated substrates. The baseline sanded surface is characterized by multiple micro-scale asperities, while the laser treated features trench-like surface patterns in the *x*-direction, *i*.*e*. perpendicular to the crack growth direction. The inserts show schematics of the locations taken for SEM imaging. Corresponding survey spectra of (**c**) Cu 2p and (**d**) O 1 s obtained using XPS analyses.

SEM images are shown in Fig. 2(a) and (b). Sanded surfaces are characterized by a random distribution of ridges and grooves that can promote an increase in surface area and roughness. Laser treated surfaces are inherently textured and provided with sub-micron scale morphological features, including characteristic sharp asperities and cavities in the recast material. The resulting interface may be able to retain the adhesive and resist debonding through micro-scale mechanical interlocking. Surface oxidation was also ascertained by means of XPS analyses. Figure 2(c) shows the survey spectrum for Cu 2p. The largest peak signal was identified to be metallic copper, although the peak is also compatible with cuprous oxide, *i*.*e*. Cu_2_O; indeed, as noted in^32^ the two compounds have very similar binding energies and are difficult to distinguish. Moreover, cupric oxide, CuO, and copper hydroxide, Cu(OH)_2_, were also identified. The appearance of the satellite peak signals corresponds to Cu^2+^ and suggests the presence of CuO. In order to investigate surface oxidation, the O 1 s survey spectra were also evaluated and are given in Fig. 2(d). The peak on the right hand side corresponds to CuO while the one on the left to Cu(OH)_2_. As a result of laser irradiation the relative intensity of the two peaks is modified and the CuO peak prevails. Copper hydroxide is presumably removed by laser irradiation. It has been reported that the formation of CuO exerts a beneficial effect on the strength of adhesive joints. It improves wettability and, in turn, increases the degree of interfacial contact with the adhesive^33^.

The surface patterning strategy proposed herein modifies the fracture energy landscape across the interface giving rise to a *toughness contrast*. Indeed, the modifications of surface chemistry and topography elevate substantially the fracture toughness. It has been shown in previous work by the authors^9^ that while the average fracture toughness of homogeneous sanded interface was equal *G*_*c,s*_ = (0.12 ± 0.03) kJ/m^2^, laser irradiation could provide an enhancement up to *G*_*c,l*_ = (2.07 ± 0.63) kJ/m^2^.

### Analysis of global responses

**Figure 3:**
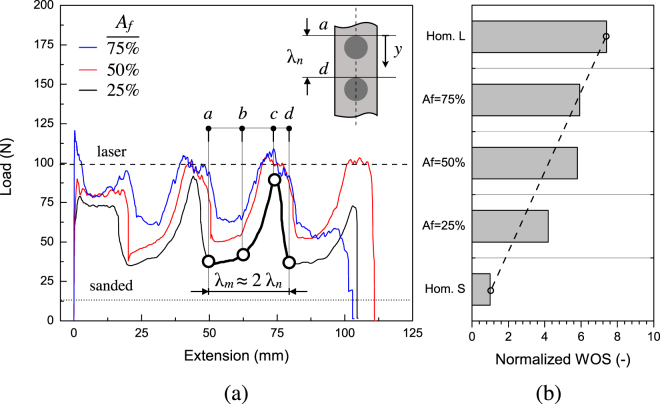
(**a**) Experimental load-displacement traces recorded in peel tests. The sudden load increase is associated to crack trapping within the laser treated area. The highlighted portion of the global response indicates a typical load fluctuation observed when the crack traverses a single period of the pattern. The insert shows the estimated initial and final position of the front. The dashed lines indicates the average peel forces of homogeneous (unpatterned) laser (top) and sanded (bottom) interfaces which were obtained by averaging the steady state values over the crack growth. (**b**) Work of separation needed to sever the samples. The dashed line show the prediction obtained using the rule of mixture. Normalization is done with respect to the energy dissipated in baseline sanded samples.

We firstly analyze the effect of area fraction of circular inclusions on global response and dissipated energy required to sever the samples. Typical steady state peeling forces *versus* applied displacement are reported in Fig. 3(a). The top and bottom dashed straight lines indicate the average steady state load measured in samples with laser treated and sanded interfaces, respectively. Both values have been determined in a previous work where the behavior of homogeneous copper/epoxy joints (*i*.*e*. no pattern) under peel loading was analyzed^9^. The fluctuation of the remote applied peel load for the patterned samples reflects the occurrence of crack *initiation*, *propagation* and *arrest* (*i*.*e*., crack pinning) which are required in order to accomplish crack growth past a laser treated area. The wavelength of the global responses (*λ*_*m*_), that is the distance between two consecutive peak or valley points, was essentially equal to twice the corresponding nominal wavelength (*λ*_*n*_) of interfacial patterns. The excellent agreement suggests the attainment of a *global* steady state peeling regime in which the opening displacement is globally (as average over one cycle) twice the crack advance^31^.

When the front approaches the tougher region the applied load increases until it reaches a peak (pinning regime). The peak force is also referred to as *effective peel force* ^14^, that is the remote load required to advance further the crack. Front pinning is reflected by the rising portion of global response ($a\mapsto c$) in which two slopes can be apparently identified and which are separated at point *b*. It is speculated that these slopes are associated to initial front pinning and subsequent crack penetration within the tougher region. The remote applied load then reaches a peak and gradually decreases before dropping suddenly to a lower bound ($c\mapsto d$). The results obtained at the lowest area fraction represent an exception since a direct abrupt load transition to a lower bound was observed.

### Effect of pattern area fraction

The results reported in Fig. 3 also show that reducing the area fraction of laser treated material the minimum load achieved within a period of crack growth decreases accordingly. It should be noted that while the minimum peel load corresponds to crack growth within the weak sanded interface, it does not match the force needed for peeling from a homogeneous sanded interface. This an indication of the occurrence of rate effects which, in turn, may depend on the spacing between consecutive inclusions, *i*.*e*. the distance *h* shown in Fig. 1(c). It is indeed in that area of the interface that, because of the toughness decrease, the crack speed increases. In particular, *h* is approximately equal to 6.5 mm at *A*_*f*_ = 25%, 3.0 mm at 50% and 0.34 mm at 75%. It is apparent that the minimum load is reduced consistently as *h* increases. It is also noted that the applied peel load drops more quickly when *A*_*f*_ decreases, *i*.*e*., when the radius of the circular features decreases. The steepest load drop ($c\mapsto d$) was observed at the lowest area fraction and it is likely associated with the excess of elastic energy which becomes available when the front is leaving the tougher interfacial region. These results suggest that the mechanism of failure shifts toward a more unstable behavior at lower *A*_*f*_ or, in other words, when the spacing between the tougher regions increases. It also suggests that the details of the global responses will be strongly affected not only by the area fraction but also by the pattern shape or distribution of laser treated material.

In order to compare the results obtained in mechanical tests, the total dissipated energy or work of separation (WOS) was elected as performance metrics. The WOS is given by the area under the force-displacement curve and includes (*i*) the micro-scale contribution arising from the fracture process zone (*W*_*f*_), *i*.*e*. breakage of the intrinsic adhesion forces and irreversible deformations/fracture of the adhesive, (*ii*) the macro-scale dissipation associated to plastic bending of copper substrates (*W*_*p*_) and (*iii*) the non-recoverable elastic energy (*W*_*e*_). The WOS is reported in Fig. 3(b) as a function of *A*_*f*_ and the quoted values are normalized with respect to the baseline sanded interface (*i*.*e*., no patterns). The WOS of patterned samples at 25% and 50% area fractions are, respectively, four and six time higher than the baseline sanded interfaces. This is a remarkable increase in the overall dissipation. However, the increase displays a non linear trend and levels out at 75%. Overall, the dissipated energy does not scale linearly with the area fraction and then does not follow the rule of mixtures (dashed line in Fig. 3).

### Effect of pattern shape

**Figure 4:**
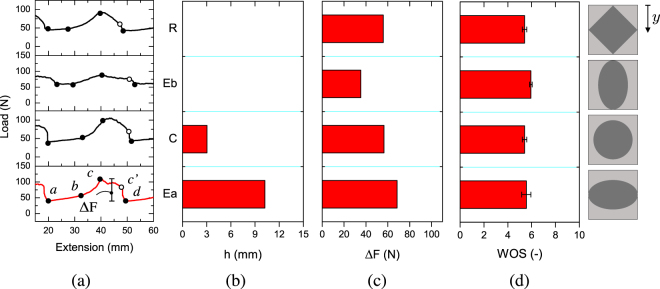
(**a**) Experimental load versus displacement curves for patterned substrates. The area fraction is constant and equal to 50%. (**b**) Distance (*h*) between the outmost points of the inclusions. (**c**) Average load fluctuations (ΔF) per period of crack propagation extracted from global responses. (**d**) Work of separation (WOS) needed to sever the samples (normalized with respect to the sanded interface).

Typical global responses associated to interfacial patterns of varying shape are reported in Fig. 4(a). The effect of pattern orientation with respect to crack propagation direction was also assessed through the analysis of the elliptical patterns since Ea and Eb have the major axis perpendicular and parallel to the direction of crack propagation (*y*-). The area fraction has been kept constant at 50% for all samples. Global responses are generally similar among each other and are all characterized by a rising branch ($a\mapsto c$) followed by a decrease and sudden load drop ($c\mapsto d$). As anticipated in previous section, the pattern geometry apparently exerts a local control of crack propagation stability since the load drop ($c^{\prime} \mapsto d$) strongly depended on the shape and the spatial arrangements of the tougher regions. Therefore, it can be interesting to compare the observed load fluctuations against the geometry of the patterns. For this reason, the distance *h* described in Fig. 1(c) as well as the experimentally determined load fluctuations (ΔF) extracted from global responses are reported in Fig. 4(b) and (c). Apparently, the obtained ΔF does not show an obvious trend with the distance *h*. Indeed, comparing the results of Ea and Eb it is inferred that ΔF increases with *h*, while comparing R and Eb, which feature *h* = 0, a significant difference in ΔF is observed. However, the extent of the load drops ($c^{\prime} \mapsto d$) depends directly on *h*. It can be also concluded that the orientation of the patterns has an important effect since the load fluctuations in Eb are pretty much lower than that displayed by Ea. The obtained results suggest that the shape and the arrangement of the patterns affect the mechanical response. In particular, the spatial distribution of tough region and the rate at which it decreases with respect to the advancing front control the propagation stability, the amplitude of load fluctuations and originates the load jumps. Finally, the WOS needed to sever the sample seems to be independent of inclusions shape as indicated in Fig. 4(d) and therefore it mostly depends on *A*_*f*_ of laser treated material.

### Finite element results

**Figure 5:**
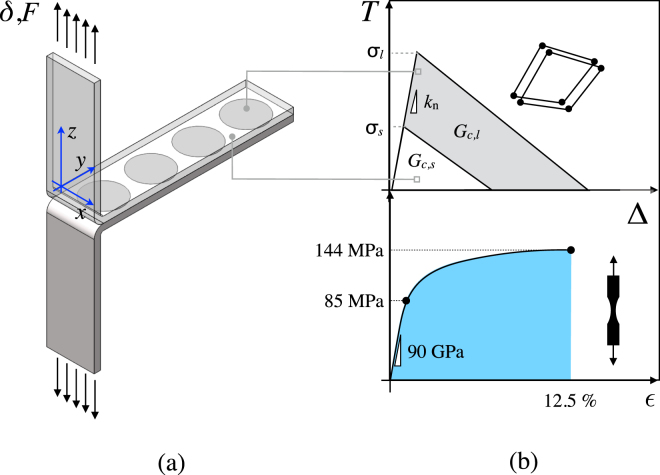
Modeling strategy undertaken to build finite element models of copper/epoxy joints. (**a**) Schematic of the T-joint highlighting the patterns across the interface. (**b**) Cohesive constitutive relationship employed to mimic the behavior of sanded and laser treated interfaces and input stress-strain response of copper substrates measured in actual experimental tensile tests.

**Figure 6:**
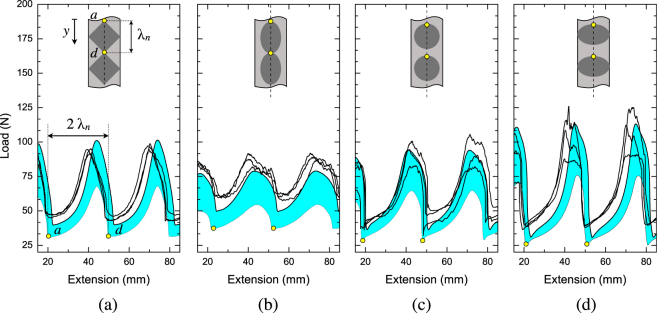
Experimental versus numerical global responses for the patterned interfaces. Results are referred to *A*_*f*_ = 50%. The shaded areas show the range of numerical predictions based on the lower and upper bounds cohesive properties of sanded and laser treated interfaces. Only portions of the global load-displacement curves are reported because of response periodicity.

**Figure 7:**
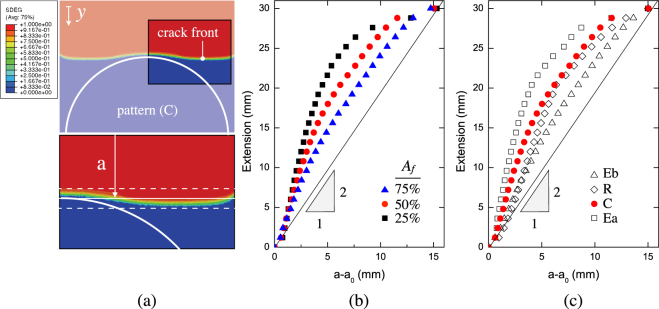
(**a**) Fringe plot obtained in finite element simulations displaying crack front pinning and the strategy employed to determine the average crack length. (**b**) Extension versus average crack advance across circular inclusions of varying area fraction. The straight line represents the linear relationship that would hold for an homogeneous interface in the case of steady state peeling regime. (**c**) Extension versus crack advance for varying pattern shapes.

Three-dimensional finite element simulations were carried out to analyze the experimental results and to ascertain the evolution of front propagation across the interface. Cohesive surfaces were used to mimic debonding within the bondline. A schematic of the model is shown in Fig. 5. It features 3D continuum elements with linear order and elasto-plastic material properties and cohesive surface elements. A bilinear cohesive constitutive relationship (*T*-Δ) was employed, with *T* being the cohesive interaction and Δ the corresponding crack face opening displacement. Fracture toughness and cohesive strength were obtained by curve fitting experimental data obtained on homogeneous sanded and laser treated interfaces (*i*.*e*., no pattern) under peel loading. Specifically, experiments and FEA were plotted simultaneously and cohesive properties were varied until the differences were negligible^9^. The corresponding set of identified cohesive properties which were used herein are: cohesive strength: *σ*_*s*_ = *σ*_*l*_ = *σ* = 29 MPa; cohesive energy: *G*_*c,s*_ = (0.12 ± 0.03) kJ/m^2^ and G_*c,l*_ = (2.07 ± 0.63) kJ/m^2^; initial stiffness: *k*_*n*_ = 50 GPa/mm. The simulated load-displacement curves for various pattern shapes and constant area fraction (*i*.*e*., 50%) are plotted against experimental measurements in Fig. 6. Only a portion of the peeling history is reported because of response periodicity. In particular, the shaded areas display the range of finite element results obtained using lower and upper bounds values of the input cohesive properties. Overall, given the complexity of the decohesion mechanism that involve crack pinning, front deflection and extensive plasticity, a reasonably good match between experiments and simulations was achieved. The global steady state peeling regime is also predicted by the finite element simulations since the wavelength of the observed load fluctuations is approximately twice the pattern spacing. In order to assess further this point, front evolution as the crack traversed the interface has been extracted from finite element simulations. The average crack length was determined as indicated in Fig. 7(a) and has been plotted against the applied displacement in Fig. 7(b) and (c). Notice that for the determination of crack length, the actual crack front was assumed to be at locations where the scalar stiffness degradation variable (SDEG) was equal to 1, *i*.*e*. no traction across the interface. The origin of the plots represents the instant at which the crack front is pinned as shown in Fig. 7(a) and which corresponds to the peel load at point *a* highlighted on the global response (cfr. Fig. 3). The straight line represents the linear scaling that would be expected for samples with homogeneous interfaces in the steady state peeling regime, *i*.*e*., the load would achieve a plateau and the applied displacement would double the crack length. However, for patterned samples, when the front traverses the laser treated region, that scaling is not linear because of crack pinning and subsequent fast propagation. The deviation from the homogeneous case scales with area fraction: the results indicate that smaller area fraction (*A*_*f*_ = 25%) delays the most the crack advance and provides efficient pinning. Moreover, for given area fraction, the shape of the inclusions also plays an important role on crack propagation. As shown in Fig. 7(c) the pattern shape Ea is the most efficient in terms of delaying crack advance. Interestingly, as shown in Fig. 4(b), this is the pattern in which the distance *h* has the highest value. This indicate once more the important effect of the spatial arrangement of the surface feature on the mechanics of crack advance across the interface.

**Figure 8:**
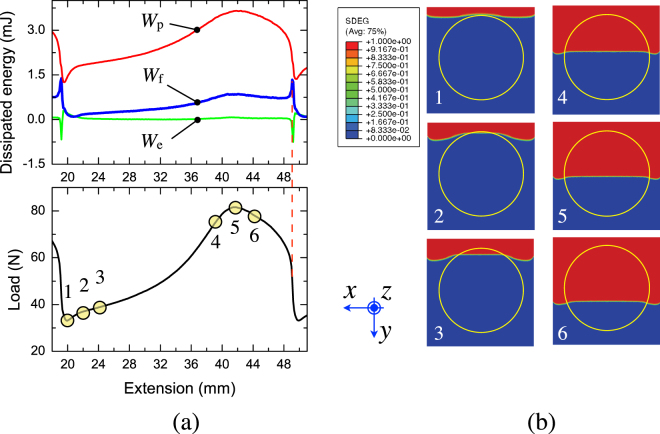
(**a**) Simulated load-extension and energy expenditure for a crack advancing within a patterned interface with circles at *A*_*f*_ = 50%. *W*_*p*_ is the plastic dissipation, *W*_*f*_ is the work of fracture and *W*_*e*_ the non recoverable elastic energy. (**b**) Analysis of crack front evolution at selected points taken from the global response displayed on the left hand side. $1\mapsto 3$: crack front pinning and subsequent penetration within the tough region. $4\mapsto 6$: crack front evolution extracted around the peak of global response. The insert shows the contour of scalar stiffness degradation (SDEG) where the blue color represents no damage (SDEG = 0) while the red color represents complete failure (SDEG = 1).

The energy dissipation associated to crack growth within one period of the pattern has been examined and the interface with circular patterns (*A*_*f*_ = 50%) has been selected to the purpose. The results are given in Fig. 8(a). By comparing the global response and the corresponding energy dissipation it is possible to infer that the stored elastic energy is negligibly small with respect to other terms, while plastic bending of the flexible adherends provides the main contribution and achieves the maximum value when the applied peel load reaches the peak value. It has been shown in previous works that the magnitude of plastic dissipation within the copper substrates is affected by the root rotation, *i*.*e*., the angle at the root of the crack, to a great extent. In particular, Kim and Aravas^34^ pointed out that there might be instances in which the measured peel force is mostly contributed by the macro-scale plastic dissipation within the sample substrates, while fracture energy of the interface represents a relatively small fraction. In order to gain further insights into the effect of patterned interfaces on energy dissipation, the crack front evolution has been extracted from finite element simulations. The results are displayed in Fig. 8(b) and are referred to the load-displacement data points highlighted in Fig. 8(a). The evolution of crack front shows the initial pinning regime followed by the lengthening of front profile and subsequent penetration within the laser treated area ($1\mapsto 3$). This is an outcome of the variable strain energy release rate associated to the toughness contrast and perimeter of the patterned region^17^. Subsequently, when the crack growths within the laser treated area, the front extends approximately to the full sample width and the remote applied loading reaches the peak value ($4\mapsto 6$). The deformed configuration of the flexible substrates corresponding to the peak peel load was extracted from finite element simulations. Careful analysis of the data indicated the occurrence of the highest root rotation when the peel load achieves the peak value.

## Discussion

The results demonstrated that the introduction of discrete areas of superior toughness heavily affects the effective peel force which oscillates as the front negotiates weak (sanded) and tough (laser) regions. In particular, sequential events of initiation, propagation and crack arrest affected the effective peel force and the overall dissipated fracture energy thereby resulting in the serrated behavior of the load-displacement responses. It is interesting to note the similarities of present experimental results with those reported in^35^, where the crack tip interaction with rigid well-bonded particles in brittle composite materials was analyzed. Like the discrete regions of superior toughness considered in this work, the presence of tough particles in a brittle solid acted as obstacles for a crack front to overcome and enhance the overall energy dissipation.

**Figure 9:**
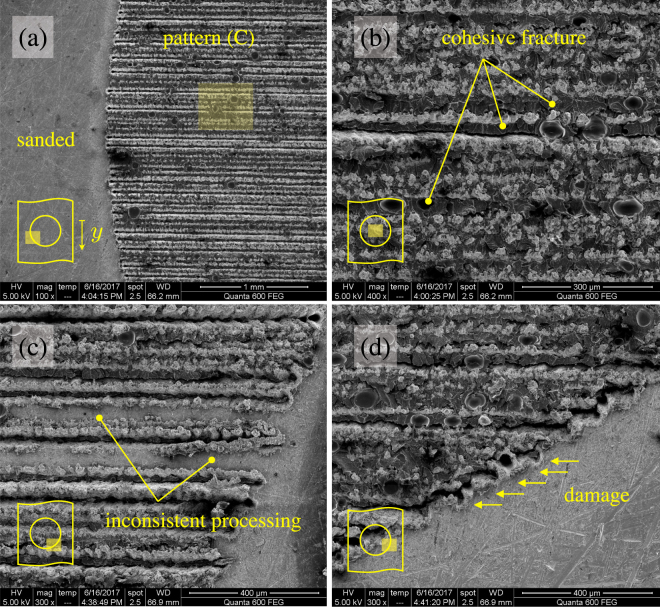
SEM images of the fracture surface. (**a**) Boundary between laser treated and sanded interfaces. (**b**) Close-up view of the area highlighted in (**a**) displaying voids and sites of cohesive failures. (**c**) Example of interfacial areas that did not receive consistent laser processing. (**d**) Typical instances of laser induced damage across the boundary of the inclusion.

The increased peel load observed in the present work enabled large plastic dissipation within the metal substrates and as a results the response of copper/epoxy joints was more ductile. Given the material system and the range of area fraction explored herein, the current technique allowed the work of fracture to increase up to six time higher than that recorded on the sanded baseline interface. Thus, the increased adhesion and toughness contrast generated through laser irradiation provided access to the large energy dissipation capacity of the plastically deforming copper substrates. In particular, the laser treated areas enabled micro-scale mechanical interlocking and cohesive failure within the adhesive layer. The SEM images of the fracture surfaces shown in Fig. 9(a) and (b) illustrate this point. Several spots of cohesive failure can be identified within the trenches induced by laser irradiation. On the other hand, the sanded interfaces displayed a brittle response and near-interfacial failure. However, care should be paid because the action of the laser beam may also induce undesired modifications and/or damage of the target material. For instance, the extreme heating/cooling rates (≈10^3^ K/s) might lead to complex metallurgical and morphological transformations of the metal and consequent embrittlement. Moreover, laser processing of relatively large areas require careful choice of the beam positioning systems. Inaccurate control of laser beam motion may lead to inconsistent laser processing, such as those highlighted in Fig. 9(c). In particular, because of inaccurate beam positioning some regions of the target material did not receive laser irradiation. Similarly, accurate selection of laser processing parameters in relation to the characteristics of the target material can have a significant impact on the quality of treated interfaces. Laser-induced damage and drilling holes, such as those shown in Fig. 9(d), may affect the integrity of the substrates and the joint reliability.

Experimental results in conjunction with finite element predictions indicated that the shape of the pattern also plays a very important role and can be used efficiently in interfacial design. Specifically, the spatial distribution of tough region controls the propagation stability, the amplitude of load fluctuations and the load jumps. This point was also highlighted by the results reported in Cuminatto *et al*.^36^ who proposed an analytical model to assess crack propagation behavior in wedge loaded DCB samples with patterned interfaces. They concluded that pattern width and spacing along the direction of crack growth affect the stability of crack propagation and the apparent toughness of the joint. Moreover, the overall crack propagation has been shown to occur in a global steady state peeling regime where the crack advance corresponds to roughly half of the applied displacement. However, the scaling varied non-linearly within a single period and the maximum applied peel load occurs when the crack is running approximately in the middle of a tough surface region. Interestingly, similar observations were also made in Xia *et al*.^12^ which carried out peel tests on patterned thin films. They considered rectangular stripe patterns (*A*_*f*_ = 25%) and, similarly to what has been reported herein, they observed that the peak peel force was achieved when the peel front was in the middle of the stripe and that the period of the oscillating force scaled with the wavelength of the stripes (*i*.*e*., global steady state fracture). However, it is recognized that in their work peel tests were carried out in the elastic range and the elevation of the peel load was only associated to the improved adhesion.

## Conclusions

In the present work pulsed laser irradiation was employed to generate patterned interfaces with enhanced adhesion properties. The strategy proposed herein was shown to be an original and very promising technique which affords the opportunity to tailor the joint behavior. In contrast to previous methods, such as template-assisted treatments, machining or molding, laser irradiation enables the tailored introduction of local toughening sites across the interface. The proposed strategy can be considered as an alternative to global/homogeneous toughening since it can reduce the manufacturing time, cost and complexity, maintain weight and structural dimensions as well as minimizing the adverse effects associated to classical toughening by the addition of a third phase. Moreover, it is quite flexible and amenable to large-scale applications where only local toughening is required at highly stressed regions. Finally, we note that the level of enhancement highlighted herein is bounded by practical factors such as the properties of the substrates. Therefore, promising avenues for future investigations include the analysis of materials with different thickness and yield stress as well as the fabrication of interfaces with random or aperiodic distribution of surface features and/or graded adhesion properties.

## Methods

### Surface treatments

Thin CuZn40 foils (100 × 100 × 0.5 mm^3^) were surface treated over a 60 × 100 mm^2^ area. Baseline samples were subjected to standard manual sanding prior to bonding (400-grit 3 M Wetordry sandpaper). Laser irradiation was carried out using a 1.06 *μ*m ytterbium fiber laser (PLS6MW Multi-wavelength Laser Platform by Universal Laser Systems, NY, USA) able to deliver a maximum pulse energy equal to 2.0 mJ and pulse duration (FWHM) equal to 10 ns. Surface modifications are generally imparted by controlling adjustable laser process parameters, such as laser power, speed (*i.e*. speed of the beam relative to the substrate), spacing (*i.e*. laser pitch) and focal distance. Based on the outcome of a previous related work carried out by the authors^9^, laser processing was herein executed 30 W average power, 30 kHz pulse frequency, 46 mm focal distance (which implied a spot size of 25 *μ*m). This choice affords the opportunity to exploit the maximum pulse fluence allowed by the available in-lab hardware (*F*_*p*_ = 200 J/cm^2^). Moreover, it was observed that a laser spacing equal to 60 *μ*m allowed the whole substrate surface to receive laser processing and to be fully treated. Laser speed was set equal to 250 mm/s.

### Analysis of surface chemistry and topography

A Scanning Electron Microscope (FEI Quanta 200) was used to analyze surface morphological modifications. Surface topography was probed using X-Ray Photoelectron Spectroscopy analyses carried out using a Kratos AXIS Ultra DLD spectrometer (KRATOS Analytical, Wharfside, UK) equipped with a monochromatic Al-K *α* anode emitting X-ray source of photon energy h *v* = 1486.6 eV. Measurements were performed at power of radiation beam equal to 150 W and the system was provided with a multichannel plate and delay line detector under a vacuum (10^−9^ mbar). Measurements were performed in hybrid mode using electrostatic and magnetic lenses. All spectra were recorded using an aperture slot of 300 *μ*m × 700 *μ*m. The survey and high-resolution spectra were collected at fixed analyzer pass energies of 160 eV and 20 eV, respectively. Samples were mounted in floating mode in order to avoid differential charging. Charge neutralization was required for all samples. Binding energies were referenced to the C 1 s peak of (C-C,C-H) bond which was set at 285.0 eV. Measurements were made at an angle of photoelectron emission of 0°, which corresponds to the average 10 nm examined thickness.

### Samples fabrication and testing

Mechanical tests were herein carried out using the T-peel test coupon^31^ whose overall dimensions are given in Fig. 1(a). Sample fabrication followed procedures similar to those reported in^9^ and therefore are herein only summarized. Copper plates were cut down into 15 × 100 mm^2^ substrates and then bonded over an area equal to 15 × 60 mm^2^ using a bi-component epoxy adhesive (Araldite 420 A/B, Huntsman, Salt Lake City, UT, USA). The bond-line thickness was maintained using 250 *μ*m thick optical fibers. After curing at room temperature for 24 hours, the adherents were bent by wedge splitting so as to confer the desired T-shape to the joint. To facilitate bending, a release film was deployed over 15 × 40 mm^2^ un-bonded region. The T-joint was pulled apart at one end using an electro-mechanical tensile testing machine (Instron 5944, Norwood, Massachusetts, USA) equipped with a 2 kN load cell; the cross-head displacement rate was set equal to 5 mm/min. Four pattern types with area fraction (*A*_*f*_) equal to 50% were engraved over the baseline sanded copper substrates. Laser scanning was made in the $x$-direction and the resulting patterns were orthogonal to the direction of crack propagation (cfr. Fig. 1(a)). The geometry of the patterns is quantified by a minimal set of parameters which are shown in the schematic of Fig. 1(b) and (c). The spatial arrangement of the features, *i*.*e*., the wavelength (*λ*_*n*_), was set equal to 15 mm for all patterns. The distance between the outmost point of consecutive features (*h*) was also obtained as described in Fig. 1(c). To explore the interaction of laser micro-machined patterns with the advancing crack front we focus on four types of pattern geometry, *i*.*e*., circles (C), ellipses (Ea and Eb) and rhombs (R). The radius *r* of each treated area as a function of the area fraction is given in Fig. 1(c). The effect of pattern orientation with respect to crack propagation direction was then assessed on the elliptical geometry, since Ea and Eb have the major axis perpendicular and parallel to the direction of crack propagation (*y*-), respectively. The area fraction of laser treated material was set equal to 50%. However, two distinct sets of samples, with circular patterns, were also included in the analysis to ascertain the effect of varying the area fraction, *i*.*e*. 25% and 75%.

### Finite element models

Three-dimensional finite element models were prepared using ABAQUS/Standard^37^. The FEA models were based on the experimental samples. The substrates were assigned 3D brick elements with linear order and elasto-plastic material properties, which were obtained through dedicated tensile tests and provided as input in tabular form. The tensile behavior was generalized to multi-axial stress state assuming isotropic hardening and using the von Mises yield surface. The adhesive layer, which supplied the resisting force during mechanical testing, was replaced by a single layer of cohesive surface elements. A bilinear cohesive constitutive relationship (*T*-Δ) was employed, with *T* being the cohesive interaction and Δ the corresponding crack face opening displacement. The basic input properties are represented by cohesive fracture energy (*G*_*c*_), cohesive strength ($\hat{\sigma }$) and initial stiffness (*k*_*n*_) which represent the area enclosed by the T-Δ curve as well as the peak stress and its initial slope, respectively. A schematic of the model, which includes bulk and cohesive elements constitutive models are given in Fig. 5. The input properties for cohesive elements were taken from previous work carried out by the authors^9^ and which addressed the behavior of homogeneous epoxy/copper interfaces (*i*.*e*. no pattern) under peel loading. Fracture toughness and cohesive strength were obtained by curve fitting, *i*.*e*., experiments and FEA were plotted simultaneously and cohesive properties were varied until FEA and experiments were indistinguishable. The following properties were then used herein: cohesive strength: *ˆσ*_*s*_ = *ˆσ*_*l*_ = *ˆσ* = 29 MPa; cohesive energy: *G*_*c,s*_ = (0.12 ± 0.03) kJ/m^2^ and *G*_*c,l*_ = (2.07 ± 0.63) kJ/m^2^; initial stiffness: *k*_*n*_ = 50 GPa/mm. A typical FE model comprised over 680000 continuum elements (C3D8R) and 55000 cohesive elements (COH3D8). The boundary conditions were such that the lower end of the sample was clamped, while the upper one was pulled in *y*-direction. The simulations were carried out in displacement control so that to closely resemble the actual experimental boundary conditions. Given the relatively large geometric deformations exhibited by the samples geometric non linearities were accounted for in the analysis. These last were carried out using an implicit finite element scheme including viscous regularization (0.01 1/s) to aid convergence.
